# Imaging Modalities for Intracranial Aneurysm: More Than Meets the Eye

**DOI:** 10.3389/fcvm.2022.793072

**Published:** 2022-02-15

**Authors:** Clémence Maupu, Héloïse Lebas, Yacine Boulaftali

**Affiliations:** INSERM, U1148-LVTS, University of Paris, Paris, France

**Keywords:** intracranial aneurysm, vessel wall imaging, imaging technique, hemodynamic imaging, inflammation imaging

## Abstract

Intracranial aneurysms (IA) are often asymptomatic and have a prevalence of 3 to 5% in the adult population. The risk of IA rupture is low, however when it occurs half of the patients dies from subarachnoid hemorrhage (SAH). To avoid this fatal evolution, the main treatment is an invasive surgical procedure, which is considered to be at high risk of rupture. This risk score of IA rupture is evaluated mainly according to its size and location. Therefore, angiography and anatomic imaging of the intracranial aneurysm are crucial for its diagnosis. Moreover, it has become obvious in recent years that several other factors are implied in this complication, such as the blood flow complexity or inflammation. These recent findings lead to the development of new IA imaging tools such as vessel wall imaging, 4D-MRI, or molecular MRI to visualize inflammation at the site of IA in human and animal models. In this review, we will summarize IA imaging techniques used for the patients and those currently in development.

Intracranial aneurysms (IA) are pathological focal dilatations of intracranial arteries mainly located at bifurcations of the circle of Willis. IAs are found approximatively in 3.2% of the adult population and are being detected mostly incidentally. Unruptured IAs are commonly asymptomatic but their rupture has severe consequences. Indeed, IA rupture leads to aneurysmal subarachnoid hemorrhage (SAH) which affects 6 in 100,000 persons per year and leads to death for 27–44% of patients (1, 2). Even if the majority of IAs do not evolve toward their rupture, 1 in 200 to 400 will (3). Therefore, there is a need to identify those IAs at risk of rupture in order to treat them and decrease this risk.

In the past few decades, several pathophysiological processes leading to IA rupture were identified as irregular IA shape, an altered hemodynamic stress within the IA and vessel wall inflammation (4). Those findings led to the development of a variety of new imaging tools which provide a better characterization of IAs and enable clinicians to identify those at risk of rupture.

This review will summarize the classical methods of imaging aneurysms and the latest development in the field. It should be noted that this review intend to provide a comprehensive overview of the imaging modalities and discuss their relevance in the field of aneurysmal pathology.

## Morphological Imaging

Imaging is a crucial diagnostic tool for the aneurysm's detection and characterization. Indeed, IA imaging can provide detailed information such as its location, size, morphology and geometry, determining the therapeutic strategy (surgical intervention or conservative management) (3). In routine clinical practice, IA are detected and imaged based on their morphology.

Digital subtraction angiography (DSA), a fluoroscopy technique using iodine contrast, is used to produce images of intracranial blood vessels without surrounding tissues as they are removed by digital subtraction (5). Thanks to its high spatial resolution, specificity and sensitivity, DSA is the gold standard for IA imaging and can determine its morphological characteristics such as its size, neck diameter and delineation (3, 6, 7). The development of 3D rotational angiography (3DRA) improved the spatial resolution of DSA, as 3D reconstruction helps to avoid imaging errors related to the superposition of vascular structures, allowing the visualization of small IAs (<3mm) (8). However DSA remains invasive and rare complications exist due to the use of intra-arterial devices and iodine-containing contrast agents during the catheter angiography [neurological: 0.1–1%; severe allergic reaction: 0.05–0.1%] (9).

Several non-invasive imaging techniques have been developed such as computational tomography angiography (CTA). CTA specificity and sensitivity are nearly as good as DSA [sensitivity for IA > 3 mm: 93.3–97.2%; specificity: 87.8–100%] (3, 6, 7). However, CTA is a poor choice for detection of small IAs localized near the skull bone as ionizing ray are almost equally absorbed by calcium and iodinated contrast agents [sensitivity = 61%] (10). Thus, a match mask bone elimination (MMBE) technique has been developed, removing non-specific signal induced by bones, but it requires a longer exposure to ionizing ray and is sensitive to patient movement (11). The advent of dual energy CTA (DE-CTA) subsequently improved material differentiation thereby reducing artifacts created by bony structures without the drawback of the MMBE method (12, 13).

Unlike CTA and DSA, magnetic resonance angiography (MRA) is performed without X-rays. MRA sequences, such as time-of-flight MRA (TOF-MRA) or non-enhanced magnetization-prepared rapid acquisition gradient echo (MPRAGE), do not require contrast agents and is thus considered the least invasive method to date. Non-contrast enhanced MRA gained interest in the last decade due to the well-known health risk of iodinated agents (14). TOF-MRA at 1.5 and 3 Tesla (T) are the most common MRA performed to visualize IAs with a greater sensitivity and accuracy for 3T [Sensitivity: 1.5T = 53.6% vs. 3T = 76.6%; accuracy: 1.5T = 84% vs. 3T = 91.9%] (15, 16). This MRA method relies on the magnetic properties of circulating blood (17). Although this allows for the elimination of contrast agents, some artifacts can be observed especially when the blood flow is turbulent or low, which constitute a limiting factor as those flow disturbances are common in large or coiled aneurysms (17, 18). To alleviate this issue, gadolinium-enhanced MRA (GE-MRA) can be performed as it is flow-independent (18–20). Both TOF- and GE-MRA have 95% sensitivity compared to DSA (6). Recently, 7T MRA has been evaluated in the study of IA. 7T MRI remains infrequent but studies agree on its high potential for the detection of IAs as well as their anatomical description and is a great tool for IA follow-up (21–23). The combination of 7T 3D-TOF and MPRAGE has been demonstrated to delineate unruptured IAs as well as DSA (22). Finally, intracranial black blood vessel imaging (MR-IBBVI), a new MRA sequence based on blood signal suppression, has been compared to TOF-MRA and DSA. Its sensitivity and specificity is higher than TOF-MRA regardless of aneurysm size [Sensitivity: MR-IBBVI = 94.5% vs. TOF-MRA = 62.7%; specificity: MR-IBBVI = 94.5% vs. TOF MRA = 92%; both compared with DSA] (24).

All these IA morphology imaging, with their benefits and disadvantages, summarized in Table 1, have a millimeter spatial resolution which is sufficient for IA detection and morphological characterization and the risk of rupture. However, vessel wall remodeling, which is a main feature of IAs evolving toward rupture, can not be observed with classical imaging mentioned above and there is currently no imaging technique to visualize the elastic lamina disruption or the thinning of the media. Optical coherence tomography (OCT), which is already widely used in ophthalmology, is being optimized for intracranial usage. OCT is based on the differential reflective properties of tissues to near infra-red light. A catheter is introduced in the targeted vessel and 2D cross-sectional images are acquired with a high resolution (1 to 15 μm) (31, 33). It has already been demonstrated that OCT allows the visualization of layers disruption in IA as the delimitations between intima and media layers are no longer visible compared to healthy vessel wall (31, 34). Moreover, the good position of intrasaccular devices can be monitored through OCT in real-time during the surgical procedure (35, 36). The development of such imaging would significantly complement the existing IA rupture risk stratification tools based on IA morphology enabled by current imaging.

**Table 1:** IA morphology imaging techniques.

**IA morphology imaging**			
**Features imaged**	**Principle**	**Observations**	**References**
**Rotational angiography (3DRA)**			
Arteries' lumen without surrounding tissues	Angiography principle: pre and post contrast rotational acquisition	- High spatial resolution; best specificity, sensitivity, depiction of small IA (<3 mm) - Iodinated agent needed - Invasive imaging (catheterization)	(5, 25)
**Computational tomography angiography (CTA)**			
**Classical CTA:** Arteries' lumen in hypersignal with surrounding tissues	Tomography principle	- Iodinated agent needed - Artifacts due to bones signal but software to remove bony structures exists - Non-invasive	(26–28)
**Dual-energy CTA**: Arteries' lumen in hypersignal with improved contrast of surrounding tissues	Same principle as classical CTA. Differs by the type of scanner used which emit X-rays of different energies	- Iodinated agent needed - Bones signal removed directly - Non-invasive	(12, 28)
**Magnetic resonance angiography (MRA)**			
**Time of flight (TOF)**: arteries' lumen in hypersignal	Principle of flow-related enhancement MRI. Under repetitive radiofrequency pulses, static tissues undergo a magnetic saturation unlike the circulating blood	- Ionizing radiation and contrast agent free - Less invasive technic - Abnormal blood flow related artifacts - Lowest spatial resolution when compared to CTA and DSA	(29)
**Gadolinium-enhanced:** arteries' lumen in hypersignal at the bolus passage	MRI sequences sensitive to gadolinium	- Ionizing radiation free - Lowest resolution when compared to CTA and DSA	(30)
**Optical coherence tomography (OCT)**			
Layers disruption in 2D-cross-sectional imaging of arteries	The differential reflective properties of tissues to near infra-red light	- Catheterization needed - High spatial resolution (μm)	(31, 32)

## Hemodynamic Imaging

All the above-mentioned imaging procedures are performed to assess the morphologic characteristics of IAs, evaluating its rupture risk. However, these parameters seem to be insufficient to accurately predict this evolution toward rupture (37). Indeed, it has been described that hemodynamics stressors are a major cause of IA formation, growth and rupture (38, 39). The main hemodynamic parameters studied are the wall shear stress (WSS defined as the frictional force tangent to vessel wall induced by blood flow), the oscillatory shear index (OSI defined as the direction and intensity flow changes during a cardiac cycle), relative residence time (RRT express the distribution of blood flow over time at the aneurysm wall) and flow patterns (40). Nowadays, a high WSS is commonly accepted to be involved in the formation of IAs, but its role in rupture is less certain as a high or a low WSS can both lead to a destructive remodeling of the aneurysm wall. Indeed, a high WSS is believed to be at the origin of a mural cell-mediated pathological response whereas a low WSS is the source of an inflammatory cell-mediated pathological response (39, 41). However, IA rupture is associated with a higher OSI, a prolonged RTT and complex flow patterns and yet, these hemodynamics parameters are not accessible *via* the current clinical imaging methods described in morphological imaging section of this review (40–43).

Computational fluid dynamics (CFD), widely used to study hemodynamic parameters, is performed on high-resolution 3D data sets (44). CFD uses static characteristics of IAs (size, location, aspect ratio, size ratio) to calculate WSS, OSI, flow velocity and RRT (39, 45). Thus, CFD is highly influenced by the choice of imaging modality, albeit no imaging modality, so far, has been described as the gold standard to CFD calculations (46, 47). Although CFD is an effective method to calculate hemodynamic parameters and has led to a better understanding of IAs, varying degrees of errors are observed due to some limitations (e.g., considers blood as a Newtonian fluid, arteries as rigid, no standardized protocol) and should be overcome in order to provide important information to clinicians (45, 46, 48).

3DRA is considered the gold standard for the detection and definition of static aneurysm characteristics, however, there are no such clear-cut opinions for dynamic features. In clinical practice, a combination of 2D and 3DRA are used to assess cerebrovascular blood flow. 2D-DSA gives flux information during the contrast-agent passage and 3DRA provides static anatomic information (49, 50). This has led to the development of 4D-DSA, also named time-resolved 3DRA, combining 2D-DSA and 3DRA. This method uses the 3D images obtained with conventional 3DRA and retains the temporal information of these acquisitions, allowing visualization of the influx and efflux of the contrast agent at any angle (51). Vanrossomme et al. reviewed several studies which successfully detected and quantified IA wall deformation between different frames with high spatial and temporal resolutions [35–165 ms and 0.2 mm] (52). Concerning hemodynamics, 4D-DSA applications have mostly been studied in arteriovenous malformations (50) and only one study assessed qualitatively the capacity of this imaging to detect IA flow pattern [excellent visualization in 27.7% of IA and fair visualization in 72.3% of IA] (53). Additionally, Lang et al. demonstrated that 4D-DSA is as reliable as 3DRA for CFD analysis as there is no significant differences in the flow velocity or WSS calculated (54). Moreover, with its high spatial resolution [voxel volume = 0.008 mm^3^] (51) 4D-DSA allows the same anatomic characterization of IAs than the gold standard 3DRA (54). Thus, 4D-DSA still needs to be improved to achieve a direct quantification of blood hemodynamics but its spatial resolution could allow, in addition to a morphological characterization of IAs, a robust CFD analysis.

Conventionally, to visualize blood flow with magnetic resonance imaging (MRI), a phase-contrast method is performed to access unidirectional flow in a 2D space (55). This 2D phase-contrast MRI has evolved to 3D time-resolved phase-contrast MRI, also called 4D-MRI. This flow imaging quantifies direct blood flow velocity in 3D, allowing flow pattern modeling and quantification of WSS, OSI and vorticity (55–58). In 2020, two complete state-of-the-art reviews on the 4D-MRI's ability to study IA hemodynamics have been published (56, 58). To summarize, 4D-MRI, mostly compared to CFD, reliably depicts intra-aneurysmal flow pattern in different IA morphologies. However, this 4D-imaging still has great limitations, in particular in terms of spatial and temporal resolution which has consequences on the calculation of hemodynamic parameters (depending on magnetic field and acquisition protocol, 4D-MRI = ranging from 0.43 × 0.43 × 0.43 to 1 × 1 × 1.6 mm^3^ voxels vs. CFD = 0.1-mm voxels) (56, 58). For instance, the WSS values had a lower magnitude when derived from 4D-MRI even if the localization of these WSS are similar (59). Another limit to the use of 4D-MRI in clinic is the long time of acquisition (depending on magnetic field and acquisition protocol, 5–30 min) (58). To overcome this limitation, an accelerated high spatiotemporal resolution 4D-7T-MRI have been proposed, providing accurate quantitative flow values with a 10 min acquisition (vs. 20 min) (60). Moreover, 4D-MRI has also been validated *in vitro* by comparing its hemodynamic measurements to those obtain by particle image velocimetry (PIV) (61). PIV is an optical imaging method which tracks particle displacement throughout a fluid field illuminated by a laser (62). As an increasingly popular *in vitro* tool to analyze fluid dynamics and validate medical flux imaging modality, PIV has been used to assess flow pattern in patient IA models with ultra-high spatial and temporal resolution [4Mpixel, 100 images/sec] (63).

Compared to classical CTA, with a longer acquisition time or several acquisitions over a given period, 4D-CTA records the influx and efflux of the contrast product and morphological changes of IA within a cardiac cycle when the acquisition is ECG-gated (64). 4D-CTA is mostly used in the evaluation of hemorrhagic/ischemic stroke and vascular malformations and has been proposed to replace the gold standard 3D-DSA for follow-up imaging since it produces accurate IA geometrics and reliable CFD results when compared to 3DRA (65).

Aside from these classical hemodynamic parameters, the notion of aneurysmal pulsatility arose. Aneurysm pulsation is an important dynamic parameter of IA since increased wall motion is assumed to be linked to a reduced stability of the aneurysm wall and, consequently, to the rupture (52, 66). This pulsation, composed of the global pulsation of the aneurysm and the movement of focal parts (blebs), must be differentiated from the physiological cerebrovascular movement during the cardiac cycle. As those pulsations are quick and of low magnitude, the development of an accurate imaging modality is a real challenge (52, 66). A study performed on 7T MRI quantifying volume pulsation showed insufficient accuracy due to multiple imaging artifacts (67). The most used imaging technique to study aneurysmal pulsation is 4D-CTA (52, 66). This imaging achieves a spatial resolution going up to the same order as the studied IA movements [high-resolution CT scans = 0.25 mm; standard scan = 0.6–0.8 mm] (52). Also, its ability to measure aneurysm pulsation in IAs larger than 5 mm *in vivo* have been reported (68). These above-mentioned imaging techniques have been summarized in Table 2.

**Table 2:** IA hemodynamics and inflammation imaging techniques.

**IA hemodynamics imaging**			
**Features imaged**	**Principle**	**Observations**	**References**
**Computational fluid dynamics (CFD)**			
Allows calculation of WSS, OSI, flow velocity and RTT	*In silico* blood flow simulation on high resolution 3D anatomical images of IAs	- Most advanced method for visualizing hemodynamic characteristics - Numerous approximations: blood as a Newtonian fluid, arteries as rigid entities	(46)
**4D digital subtraction angiography (4D-DSA)**			
Influx and efflux of the contrast product and therefore of the blood flow pattern and arteries' lumen in hypersignal	Same principle as 3DRA. Differs in the images processing	- As reliable as 3DRA for CFD analysis - High spatial resolution - Iodinated agent needed - Most invasive imaging (catheterization)	(50, 51)
**4D-magnetic resonance angiography (4D-MRA)**			
Characterization and quantification of WSS, blood flow pattern and velocity	Principle of a flow-sensitive MRI (Phase contrast-MRI). Under bipolar gradient, blood emit a signal directly proportional to its speed	- Direct quantification of blood flow velocity - No contrast agent - Long time acquisition	(55, 56)
**4D-computational tomography angiography (4D-CTA)**			
Blood flow pattern and arteries' lumen in hypersignal	Same principle as classical CTA. Differs in protocol of acquisition to have temporal information	- Promising technic to study aneurysm pulsation - Longer exposition to ionizing ray compared to CTA	(64)
**IA inflammation imaging**			
**Macrophage imaging**			
Inflamed arteries' wall in hyposignal	Property of ferumoxytol to be engulfed by macrophages and detectable using specific MRI sequences	- Risk of allergic reaction to ferumoxytol - Technically difficult and time consuming	(69, 70)
**Vessel wall imaging (VWI)**			
Inflamed arteries' wall in hypersignal	MRI sequences which suppress both blood and cerebrospinal fluid signal	- High negative predictive value; moderate positive predictive value - Stagnant flow artifact - Lack of reproducibility	(71, 72)

## Inflammation Imaging

Over the past decades, a growing amount of evidence seems to involve vessel wall inflammation in the pathogenesis of IA (73). Indeed, several histological studies demonstrated that inflammatory cells (mainly T-cells and macrophages) infiltration and complement activation are associated with IA rupture (74, 75). In line with this observation, vessel wall inflammation detection could help to identify IA at high risk of rupture. In order to develop new tools to visualize inflammation *in vivo*, non-invasive inflammation imaging has been developed over the past few years.

As macrophage infiltration is a typical feature observed in aneurysmal vessel wall, macrophage imaging emerged thanks to the development of ferumoxytol. Ferumoxytol is a superparamagnetic form of iron oxide, engulfed by macrophages and detectable using MRI. Therefore, MRI after ferumoxytol infusion can reflect macrophage activity and associated inflammation within aneurysmal vessel wall. A first histological study analyzed unruptured aneurysm tissues from patients displaying ferumoxytol-induced hyposignal (72h after ferumoxytol infusion) and observed both macrophage infiltration and iron particle uptake by IA vessel wall (69). Intriguingly, they observed a noticeably different level of ferumoxytol uptake among patients, some considered with “early uptake” (visible 24h after infusion) or “late uptake” (visible 72h after infusion) (76). The authors showed that IA with “early uptake” had a similar level of macrophage infiltration compared to ruptured IA, and it was significantly higher in “early uptake” IAs vs. “late uptake” IAs. Finally, all of the “early uptake” IAs managed conservatively evolved to rupture within 6 months while no “late uptake” IAs did. Thus, this study by Hasan et al. suggested that ferumoxytol-MRI could identify unstable IAs at high risk of rupture within 6 months. However, since iron is abundant in red blood cells, subtraction of pre- and post- ferumoxytol infusion images is required to detect ferumoxytol engulfed by macrophages. These pre- and post- infusion images performed independently make this analysis technically difficult and time-consuming therefore, a simpler diagnostic method is desirable (70, 77).

Vessel wall imaging (VWI) has recently emerged as a promising diagnostic tool to image intracranial vessel wall inflammation through MRI. This technique, also known as black blood MRI, provides only signals from the vessel wall thanks to the suppression of both blood and cerebrospinal fluid signal (CSF). The acquisition of VWI demands high resolution, therefore a 3T or higher magnet strength is required. Briefly, VWI generally consist in T1-weighted pre- and post- contrast sequences along with blood and CSF suppression obtained with a 3D turbo spin-echo sequence with variable flip angle refocusing pulses (71). VWI sequence names differ among MRI constructors: VISTA (volume isotropic turbo spin-echo acquisition; Phillips healthcare, Eindhoven, Netherlands), SPACE (sampling perfection with application-optimized contrasts by using different flip angle evolutions; Siemens Healthinners, Erlangen, Germany) or CUBE (GE Healthcare, Milwaukee, WI, USA) (78).

Thanks to blood signal suppression, VWI has been used to study aneurysm wall structure, thickness and wall enhancement. Aneurysm wall enhancement (AWE) is mainly qualitatively assessed and can be classified as focal or circumferential. Radio-histological correlation studies revealed that focal AWE (FAWE) is associated with fresh intraluminal thrombus at the rupture site (79). This finding can provide useful information for the surgical treatment of ruptured IA before treating the patient by microsurgical clipping or endovascular coiling. FAWE can also be observed in unruptured IA and colocalized with hemodynamic factors in favor to a higher rupture risk (80). Moreover, FAWE is observed in areas of morphological changes in the IA vessel wall, supporting the hypothesis that FAWE could be a marker of instability (80). On the other hand, circumferential AWE (CAWE) is thought to be due to wall thickening with abundant inflammatory cell infiltration and neovascularization (78, 81, 82).

In cases of subarachnoid hemorrhage and multiple aneurysms, several criteria are used to determine which one underwent rupture (*i.e*. hemorrhage localization, IA size, location, shape, aspect ratio). As vessel wall inflammation is a risk factor of IA rupture, AWE is nearly always observed in ruptured IAs (83). Along with this observation, some studies performed on patients presenting multiple IAs, demonstrated that VWI can identify the ruptured IA which is characterized by a thick vessel wall enhancement (84, 85). Thus, VWI can be a useful diagnostic tool in identifying ruptured IA and its site of rupture (79).

With regard to unruptured IA, current research is deciphering the clinical interpretation of AWE. It has been proposed that AWE could be a biomarker of vessel wall inflammation in unstable IAs prone to evolve toward rupture (86). Indeed, some studies performed on unruptured IAs demonstrated a correlation between AWE and common risk factors of IA rupture such as a larger size (≥7 mm), an irregular shape, a high aspect ratio (depth/neck width) and its localization in the anterior cerebral, posterior communicating and posterior cerebral arteries (87–91). A correlation between AWE intensity and the severity of PHASES and ELAPSS scores has also been demonstrated (92).

Finally, a meta-analysis performed on 6 studies analyzed VWI and aneurysm instability. The authors concluded that unstable aneurysms (defined as ruptured, symptomatic, or growing on serial imaging) had statistically higher odds to display AWE. There was still a significant correlation between AWE and IA instability after the removal of ruptured aneurysms (93). Moreover, these meta-analyses highlighted that the absence of wall enhancement on VWI is strongly associated with IA stability (negative predictive value: 96.2%). Very recently, another meta-analysis added 6 more studies, including a longitudinal prospective study, and confirmed these positive and negative predictive value (94, 95). Therefore, VWI and AWE could be a useful risk stratification tools in assessing IAs stability.

Despite potential clinical applications of VWI, it is important to highlight potential limitations of this new diagnostic tool. The meta-analysis demonstrated a moderate specificity (62.7%) and positive predictive value (55.8%) of AWE in identifying unstable aneurysms, meaning that a part of IAs with AWE on VWI are considered to be stable (93). Moreover, flow artifacts within the sac, contrast extravasation and stagnant flow could mimic AWE, leading to false-positive signals (96, 97). In addition, there is no consensus on the definition of AWE as some studies included both FAWE and CAWE whereas others only studied CAWE. Most studies qualitatively assessed AWE inducing a lack of reproducibility, therefore quantitative AWE measurement should be considered (91). Finally, there is a heterogeneity concerning the definition of unstable IA qualified as growth, morphology changes, symptomatic and/or rupture (97). The different inflammation imaging methods have been summarized in Table 2.

### Imaging in Animal Model of IAs

Understanding of IA pathophysiology has been largely enabled by the use of small animal models (rat, mouse, rabbit) in which induced IA can mimic aspects of the human pathology. As such, induced IA are smaller than those found in humans thus, the above-mentioned imaging techniques are not widely used to analyze IA's dynamic and static characteristics (98). In fact, only a few studies use them other than as IA detection tools, for instance to detect macrophage infiltration or to perform CFD analysis on 3DRA images (77, 99). To the best of our knowledge, only 2 studies managed to follow aneurysmal remodeling in mice over 3 months using really high-field MRI (9,4T) (100, 101). Regarding IA's hemodynamics, Doppler ultrasound imaging can be used to measure blood flow velocity in rabbit models of internal carotid aneurysm (99, 102, 103).

## Conclusion and Perspectives

As IA pathophysiology becomes better understood, new factors contributing to IA progression and rupture are discovered, such as altered hemodynamic parameters or inflammation within IA vessel wall. Novel imaging technique must be developed to visualize these important characteristics and provide essential information's to clinicians for a better IA management. One can speculate that the combination of different imaging techniques that rely on morphological, hemodynamic and inflammatory markers will allow clinicians to accurately assess the risk of aneurysm rupture and adopt the best care strategy.

## Author Contributions

HL and CM did the literature research and wrote the first draft of the review. YB, HL, and CM contributed to the idea of the manuscript. YB provided critical feedback. All authors reviewed the manuscript and approved the submitted version.

## Funding

This work was funded by a predoctoral fellowship from the Doctoral School Hématologie – Oncogenèse – Biothérapies ED 561 (to CM) and by the European Research Council grant 759880 (to YB).

## Conflict of Interest

The authors declare that the research was conducted in the absence of any commercial or financial relationships that could be construed as a potential conflict of interest.

## Publisher's Note

All claims expressed in this article are solely those of the authors and do not necessarily represent those of their affiliated organizations, or those of the publisher, the editors and the reviewers. Any product that may be evaluated in this article, or claim that may be made by its manufacturer, is not guaranteed or endorsed by the publisher.

## Abbreviations

AWE: Aneurysm wall enhancement
CAWE: Circumferential aneurysm wall enhancement
CFD: Computational fluid dynamics
CSF: Cerebrospinal fluid signal
CTA: Computational tomography angiography
DE-CTA: Dual energy computational tomography angiography
DSA: Digital subtraction angiography
FAWE: Focal aneurysm wall enhancement
GE-MRA: Gadolinium-enhanced magnetic resonance angiography
IA: Intracranial aneurysm
MMBE: Match mask bone elimination
MPRAGE: Magnetization-prepared rapid acquisition gradient echo
MRA: Magnetic resonance angiography
MRI: Magnetic resonance imaging
MR-IBBVI: Magnetic resonance- intracranial black blood vessel imaging
OCT: Optical coherence tomography
OSI: Oscillatory shear index
PIV: Particule image velocimetry
RRT: Relative residence time
SAH: Subarachnoid hemorrhage
T: Tesla
TOF-MRA: Time-of-flight magnetic resonance angiography
VWI: Vessel wall imaging
WSS: Wall shear stress
3DRA: 3-dimensional rotational angiography.
